# Putting the brakes on acute lung injury: can resolvins suppress acute lung injury?

**DOI:** 10.3389/fphys.2012.00445

**Published:** 2012-11-29

**Authors:** Ruan R. Cox, Oluwakemi Phillips, Narasaiah Kolliputi

**Affiliations:** Division of Allergy and Immunology, Department of Internal Medicine, Morsani College of Medicine, University of South FloridaTampa, FL, USA

**A commentary on**

**Aspirin-triggered resolvin D1 reduces mucosal inflammation and promotes resolution in a murine model of acute lung injury**

by Eickmeier, O., Seki, H., Haworth, O., Hilberath, J. N., Gao, F., Uddin, M., et al. (2012). Mucosal Immunol. doi: 10.1038/mi.2012.66

Acute lung injury (ALI), a syndrome of respiratory failure, is a major clinical problem in the United States. With a high incidence rate, affecting nearly 200,000 annually and a significant morbidity and mortality rate, ALI represents a significant source of health care expenditure with a cost of 3.5–6 billion dollars annually (Treggiari et al., 2004; Rubenfeld et al., 2005; Raghavendran et al., 2011). Clinically, ALI presents as a decrease in the partial pressure of oxygen per fraction of inspired oxygen without the presence of left arterial hypertension (Tomashefski, 2000). ALI is also characterized by pulmonary edema, deterioration of the alveolar-capillary membrane and an aggressive inflammatory response. Acute insults that affect the continuity of the alveolar-capillary membrane, such as aspiration of gastric content, sepsis, and hyperoxic therapy lead to ALI (Dos Santos and Slutsky, 2006; Kolliputi et al., 2010). The alveolar membrane is an intricate system that not only regulates gas exchange, but also provides a complex defense mechanism against foreign particles and organisms. Pneumocytes, unique cells in the alveolar epithelium, are responsible for facilitating gas exchange, regulating fluid transport, and secreting surfactant to reduce alveolar surface tension. When the alveolar barrier is disrupted, proteinaceous exudates and extracellular components of necrotic pneumocytes activate resident alveolar macrophages causing massive cytokine release (Ware and Matthay, 2000). The neutrophilic inflammation and blunted gas exchange that results requires aggressive medical care. The inflammatory response, if left uncontrolled, can lead to further deterioration of the lung epithelium and the development of a fibroproliferative environment (Raghavendran et al., 2011). While many researchers have focused on the molecular determinants of inflammation, the biochemical pathways detailing the resolution mechanism have not been thoroughly investigated.

In the July 2012 issue of *Mucosal Immunity*, Eickmeier et al., discuss the presence of resolvins, proresolving lipid mediators, and present exciting findings on their role in the natural resolution of ALI (Eickmeier et al., 2012). Resolution phase interaction products (resolvins) are omega-3 polyunsaturated fatty acid derivatives of potent anti-inflammatory precursors, eicosapentaenoic acid (EPA), and docasahexaenoic acid (DHA) (Serhan et al., 2002). “E-series” and “D-series” resolvins are derived from EPA and DHA, respectively. The airway mucosa has been shown to be rich in DHA (Freedman et al., 2004), however, the conversion of DHA to D-series resolvins has not been shown. Resolvin D1 (RvD1), a derivative of DHA, has been found in murine resolving inflammatory peritoneal exudates (Serhan et al., 2002). To investigate the potential role that RvD1 may play in the resolution of ALI, Eickmeier et al. used a murine aspiration pneumonitis acute lung injury (APALI) model induced by hydrochloric acid (HCl) administration into the left lung. Picogram quantities of RvD1 were found using metabolipidomics analysis following HCl instillation. Immunohistochemical analysis also showed enhanced expression of RvD1 receptor (ALX/FPR2) as early as 2 h post-APALI. This suggested that there indeed was a conversion of DHA to RvD1 following lung injury. Activation of ALX/FPR2 has previously been demonstrated to dampen the inflammatory responses through blockage of proinflammatory MAP kinase and NF-κB signaling (Chiang et al., 2006). Utilizing an intra-venous administration of the more stable form of RvD1, aspirin-triggered resolvin D1 (AT-RvD1), Eickmeier et al. demonstrated that the conversion of DHA in pulmonary mucosa alleviates the effects of inflammation in APALI. AT-RvD1 showed therapeutic effects when given prior to or post HCl instillation. Bronchio-alveolar lavage fluid (BALF) collected from AT-RvD1 treated mice contained decreased leukocytes and proinflammatory cytokines in comparison to control. AT-RvD1 treated mice demonstrated decreased lung resistance and improved lung mechanics in comparison to controls, a result that was correlated to enhanced epinephrine secretion in BALF. Utilizing conventional methods of wet-to-dry ratio, Evans blue dye BALF content, and FITC-dextran serum content analysis, the authors showed that AT-RvD1 restored barrier integrity in APALI mice in comparison to control. As mentioned previously, the anti-inflammatory effects of ALX/FPR2 activation were shown to be a result of reduced activation and nuclear translocation of the transcription factor NF-κB. Eickmeier et al., demonstrated that mice treated with AT-RvD1 demonstrated reduced NF-κB phosphorylation, which is necessary for the activation, translocation and DNA binding functions of this proinflammatory molecule.

The work of Eickmeier et al. revealed that RvD1 is a central mediator in the endogenous attenuation of inflammation seen in APALI. In most cases of ALI, the injury is indeed self-limiting and resolves on its own (Dos Santos and Slutsky, 2006). This work is particularly important because it gives insight to the mechanism involved in the lung injury resolution process. A recent clinical study demonstrates that, ALI progression is associated with increased ventilator time and longer intensive care unit (ICU) stays. These patients show an enhanced proinflammatory cytokine profile which was also correlated with increased morbidity (Dolinay et al., 2012). Previous reports have also demonstrated that ALI/ARDS patients represent 34% of yearly costs for all ICU trauma patients (Treggiari et al., 2004). In the case that the ALI does not resolve, the patient is at risk for developing acute respiratory distress syndrome in as little as 3 days (Marshall et al., 1998). Finding endogenous mediators that may control the ungoverned inflammation seen in ALI is a pivotal step to finding a treatment for this disease that entails more than just supportive care (Marshall et al., 1998). Because of the self-limiting nature of the APALI model, as well as the heterogeneously diffuse causes that may lead to ALI, the ability of resolvins to resolve ALI caused by other risk factors and insults needs to be investigated. Evaluating the effect of resolvins on other forms of ALI represents a crucial next chapter in the search for a cure to this debilitating disease. Furthermore, the work of Eickmeier et al. has paved the way for the exploration of the beneficial effects of resolvins in the incidences of other sterile injuries, such as atherosclerosis, gout, Alzheimer's disease, and diabetes. Hopefully these findings will lead to the resolution of the war that we wage in our bodies under inflammatory conditions.
